# Coliphages of the human urinary microbiota

**DOI:** 10.1371/journal.pone.0283930

**Published:** 2023-04-13

**Authors:** Elias Crum, Zubia Merchant, Adriana Ene, Taylor Miller-Ensminger, Genevieve Johnson, Alan J. Wolfe, Catherine Putonti

**Affiliations:** 1 Bioinformatics Program, Loyola University Chicago, Chicago, Illinois, United States of America; 2 Department of Biology, Loyola University Chicago, Chicago, Illinois, United States of America; 3 Department of Microbiology and Immunology, Stritch School of Medicine, Loyola University Chicago, Maywood, Illinois, United States of America; University of Texas at San Antonio, UNITED STATES

## Abstract

Due to its frequent association with urinary tract infections (UTIs), *Escherichia coli* is the best characterized constituent of the urinary microbiota (urobiome). However, uropathogenic *E*. *coli* is just one member of the urobiome. In addition to bacterial constituents, the urobiome of both healthy and symptomatic individuals is home to a diverse population of bacterial viruses (bacteriophages). A prior investigation found that most bacterial species in the urobiome are lysogens, harboring one or more phages integrated into their genome (prophages). Many of these prophages are temperate phages, capable of entering the lytic cycle and thus lysing their bacterial host. This transition from the lysogenic to lytic life cycle can impact the bacterial diversity of the urobiome. While many phages that infect *E*. *coli* (coliphages) have been studied for decades in the laboratory setting, the coliphages within the urobiome have yet to be cataloged. Here, we investigated the diversity of urinary coliphages by first identifying prophages in all publicly available urinary *E*. *coli* genomes. We detected 3,038 intact prophage sequences, representative of 1,542 unique phages. These phages include both novel species as well as species also found within the gut microbiota. Ten temperate phages were isolated from urinary *E*. *coli* strains included in our analysis, and we assessed their ability to infect and lyse urinary *E*. *coli* strains. We also included in these host range assays other urinary coliphages and laboratory coliphages. The temperate phages and other urinary coliphages were successful in lysing urinary *E*. *coli* strains. We also observed that coliphages from non-urinary sources were most efficient in killing urinary *E*. *coli* strains. The two phages, T2 and N4, were capable of lysing 83.5% (n = 86) of strains isolated from females with UTI symptoms. In conclusion, our study finds a diverse community of coliphages in the urobiome, many of which are predicted to be temperate phages, ten of which were confirmed here. Their ability to infect and lyse urinary *E*. *coli* strains suggests that urinary coliphages may play a role in modulating the *E*. *coli* strain diversity of the urobiome.

## Introduction

While several different bacterial species are known to cause urinary tract infections (UTIs), uropathogenic *Escherichia coli* (UPEC) is estimated to account for up to 74.4% of all community-acquired infections [1]. The presence of *E*. *coli* is often taken as evidence of UTI. While UTIs can be the result of colonization of the urinary tract by *E*. *coli* strains from the gut [2–5], *E*. *coli* can also be a member of the urinary microbiota (urobiome) of individuals without lower urinary tract symptoms (LUTS) [6–8]. In fact, recent studies found that *E*. *coli* can be the predominant taxon in female without LUTS, particularly in some older females [8, 9]. Furthermore, human host genetic make-up can contribute to the presence of *E*. *coli* in the urine of females without UTI symptoms [9].

The urobiome of individuals with or without LUTS is home to a wide variety of other bacterial taxa (see reviews [10–13]), fungi [14], and viruses [15]. In fact, viruses are the most abundant members of the urobiome and recently have been associated with LUTS [16]. Viruses that infect bacteria (bacteriophages or phages) far outnumber human viruses in the urobiome [15, 17, 18]. Similar observations have been made in other organs’ microbiota [19]. Phages can drive bacterial diversity within a community through predation [20–22]. Phages that integrate into their bacterial host’s genome (prophages) can increase the virulence of their host [23, 24], e.g., by encoding for toxins [25, 26]. While studies of phage-bacteria dynamics have yet to be conducted for the urobiome, investigations in the gut microbiota are ongoing [27].

Prior studies of urinary bacterial genomes found that most strains harbor one or more prophage sequences [28–30]. This includes bacterial species associated with urinary health, e.g., *Lactobacillus* species [28, 29], as well as bacterial species associated with UTIs, e.g., *E*. *coli* [7, 28] and *Proteus mirabilis* [28]. These phages replicate with their bacterial host and are either integrated in the bacterial host genome or persist as an extrachromosomal plasmid; this is referred to as the lysogenic life cycle of the phage. Our previous work with urinary strains harboring prophages found that many of these prophages could switch from the lysogenic life cycle to the lytic (predatory) life cycle [28, 30–32]. This switch, a process called induction, is mediated by intrinsic and/or extrinsic factors that cause the prophage to excise itself from the bacterial genome, replicate to produce mature (lytic) phages, and burst or kill the host cell (see review [33]). Bladder-relevant stressors, e.g., changes in pH, have been shown to be effective in inducing prophages [31].

Prophages and phage genes have been routinely identified in urinary *E*. *coli* genome sequences [28, 34–37]. While phages and prophages that infect *E*. *coli* (coliphages) in the lab have been studied for decades [38] and coliphages from urine samples have been isolated and characterized [39–42], coliphage diversity within the urobiome has yet to be thoroughly investigated. Here, we present the first catalog of urinary *E*. *coli* prophages. All publicly available urinary *E*. *coli* genomes were examined for prophage sequences and the diversity and genic content of these prophages was explored. Ten urinary prophages were then induced from urinary *E*. *coli* strains, and we assessed their ability to lyse laboratory and urinary *E*. *coli* strains isolated from females with UTI or overactive bladder (OAB) symptoms. Additionally, we tested several urinary coliphages and laboratory coliphages against these same set of urinary *E*. *coli* strains. We find that coliphages are ubiquitous in the urobiome, including temperate coliphages capable of lysing other urinary *E*. *coli* strains.

## Methods

### Prophage identification

All publicly available *E*. *coli* complete and draft genome assemblies in NCBI that were documented as being collected from urine or the urinary tract in the genome metadata were downloaded (February 2021). 906 genomes were obtained from NCBI meeting this criterion; 2 sequences were removed due to quality concerns. The final set of 904 *E*. *coli* sequences (**S1 Table**) were examined using PHASTER [43]. A Python script was written to pull results from the PHASTER API and to separate PHASTER predicted “intact,” “questionable,” and “incomplete” prophage sequences. This script is available at https://github.com/putonti/phaster_commands.

All intact prophage sequences were compared via local blastn queries to a database of all complete and partial phage genome sequences in GenBank as of February 2021 (Advance Query Fields—Organism: “Virus” and Division: “PHG”). This database includes 26,381 sequences. Results with a query coverage greater than 50% and a percent identity greater than 70% were considered high confidence hits and the associated taxonomies of the GenBank phage records were used to predict the taxonomies of the predicted urinary phages.

Furthermore, all intact prophage sequence were screened for antibiotic resistance genes using the RGI tool v.5.2.1, which uses CARD [44], exploring perfect, strict, and loose hits, and virulence factors via the Virulence Factor Database (VFDB) [45]. RGI was installed locally using conda, and the CARD database (v.3.1.4) was downloaded. The RGI program was run using default parameters. For virulence factor screening, the full dataset of known bacterial virulence factor gene sequences was downloaded from VFDB on March 2021 (http://www.mgc.ac.cn/VFs/download.htm). These sequences were made into a local database via the makeblastdb command (BLAST+ v.2.9.0). Prophage sequences were queried against this database using BLASTn with the parameter -evalue 0.001. Hits of <90% sequence identity and query length <90% of the virulence factor gene sequence were removed from further consideration. The virulence factor gene descriptions were determined using VFDB.

The PATRIC online tool was used to annotate the intact prophage sequences with the “Annotation Recipe” parameter set to “Bacteriophage” [46]. All annotated genes containing “integrase” in the description were added to a multi-FASTA file, and Kalign v.2.04 was used to produce a multiple sequence alignment with default parameters [47]. The multiple sequence alignment was manually inspected using Geneious Prime 2021.2.2 (Dotmatics, Auckland, NZ). Partial integrase gene sequences were identified through this manual inspection and were removed from the data set. The remaining integrase gene sequences were aligned again with Kalign. This alignment aided in confirming the identity of integrase genes within the predicted prophage sequences.

## Prophage network construction

Using Anvi’o v.6.2 [48], the intact prophage sequences were annotated, and a coliphage pan-genome was constructed. Prophage sequences were made into an Anvi’o database annotated using the ‘anvi-run-hmms’ command. The annotated prophage sequences were then used to produce a coliphage pan-genome using the Anvi’o ‘anvi-pan-genome’ command with an mcl-inflation of 2 and a minbit of 0.35 to identify homologous genes among the prophage sequences.

An R script (www.R-project.org) was written to derive a network of prophages. The methods described here for phage network construction were adapted from our prior work [49]. Using the output result (mcl-cluster.txt) of the Anvi’o mediated clustering using the Markov Clustering Algorithm (MCL), the MCL results were translated into a genome-gene presence/absence matrix, *P*, in which each entry {*i*,*j*} was 1 if virus genome *i* contained a homolog found in gene cluster *j*. This matrix is equivalent to the adjacency matrix for a bipartite network of phage genomes and genes. Adjacency matrices for the genome and gene level networks were then created as *A*_genome_ = sign(*P* × *P*^*T*^) and *A*_gene_ = sign(*P*^*T*^ × *P*), where *T* indicates the matrix transpose. The sign() function replaced all nonzero entries resulting from the original matrix products with a 1, converting the matrices from weighted to unweighted adjacency matrices. These matrices were then transformed into undirected graphs and corresponding edge lists using igraph (https://igraph.org/). Thus, for the genome-level network, two genomes are considered connected if they share any genes. The connections were filtered using a normalization calculation: $w=(\#\;\text{of}\;\text{shared}\;\text{genes}\;\text{between}\;\text{genomes}\;1\;\text{\&}\;2)/\sqrt{\left(l_{1}\times l_{2}\right)}$, where *l*_*i*_ is the size of the genome *i*. By designating a minimum value of *w* (*minw*) that allows for an edge to be drawn between two genomes only if *w* > *minw*, the edges were filtered to construct networks of differing connectivity.

The edge-lists constructed from the edge-drawing Rscript were then visualized using Cytoscape [50]. Different values of *minw* were considered for the PHASTER predicted prophages.

### Prophage sequence clustering

All intact prophage sequences were clustered using cd-hit-est v.4.6 [51, 52]. The following parameters were used: sequence identity threshold = 80% (0.8), length of difference cutoff = 80% (0.8), word length = 4. The “accurate but slow mode” algorithm was used. For each cluster, cd-hit-est selected one sequence as a representative sequence (indicated by “*” in the output .clstr file). For more details regarding the process implemented by CD-HIT algorithms for selecting representative sequences, see Huang et al. [53]. Sequences of representatives of each cluster are available upon request.

### Comparison of urinary coliphages to gut phageome

All phage sequences from metagenomic data sets from the gut microbiome were retrieved from the mMGE database [54] (accessed July 2021). Intact urinary prophage sequences were compared against this database using a local blastn query. For these results, our thresholds were query coverage ≥ 50% and sequence identity ≥ 70%, although all results meeting the query coverage threshold had a sequence identity ≥ 80%.

### Prophage induction

Nine strains of *E*. *coli* isolated from urine samples were used for induction experiments. The urinary strains were selected based upon their genome analysis by PHASTER [43]; all 9 were predicted to contain at least one intact prophage sequence. For pH-based induction, we used the previously described protocol [31]. Briefly, urinary *E*. *coli* strains were grown overnight in LB at 37°C with shaking. These strains include *E*. *coli* UMB0527, UMB6653, UMB6721, UMB9006, UMB9105, UMB9208, UMB9344, UMB9346, and UMB9930. While UMB9006 was obtained from a “clean-catch” voided urine sample, the remaining 8 samples were isolated from catheterized urine samples. These strains were obtained through prior IRB-approved studies (Loyola University Chicago: LU204195 and LU209545, and University of California San Diego: 170077AW) as part of prior separate studies [55, 56]. The overnight culture was then subcultured into 3 mL of LB adjusted to different pH values, pH = 4, 7, and 9 and grown overnight at 37°C with shaking. These pH values were selected informed by our prior work [31]. These pH-adjusted cultures were filtered using a 0.22um CA syringe filter. Filtrate was next spotted onto lawns of naïve laboratory strains of *E*. *coli* B, *E*. *coli* C, and *E*. *coli* K-12. For each lawn, 500 uL of turbid (overnight) *E*. *coli* culture + 3 mL of soft (0.7%) LB agar were mixed and spread atop a 1.7% LB agar plate. The spot plates were then incubated overnight at 37°C. Plaques were harvested and used to reinfect the laboratory strain, i.e., the bacteria of the lawn from which the plaque was harvested, and incubated again overnight at 37°C. These cultures were filtered as previously described and plated using the pour plate technique (100 uL of phage lysate + 500 uL of turbid *E*. *coli* culture + 3 mL of soft LB agar); a single plaque was picked, suspended in LB, vortexed, filtered, and added to turbid *E*. *coli* overnight cultures. This process was repeated at least 3 times to plaque purify the phage and subsequently to create higher titer stocks of the induced urinary prophage.

Prophage identification was performed by PCR. Primers were designed using Primer-BLAST [57] to amplify the PHASTER predicted intact prophage sequences. A primer pair was designed for each individual predicted intact prophage sequence; thus, a strain harboring multiple predicted intact prophage sequences would have multiple pairs (**S2 Table**). Preference was given to primers that amplified coding regions with a predicted protein function, i.e., not hypothetical proteins. All predicted prophage sequences were annotated using the RAST server [58] and visualized using Geneious Prime. If more than one intact prophage was predicted for a given strain, primers were designed for each predicted prophage. Primers were synthesized by Eurofins Genomics LLC (Louisville, KY USA). The original bacterial strain was used as a positive control for PCR reactions of its respective induced prophages. Amplification was confirmed via agarose gel. The PHASTER predicted sequences were queried online against the nr/nt viruses (taxid:10239) to identify the closest related sequenced phage.

### Phage host range

Urinary and laboratory *E*. *coli* strains were grown in LB overnight at 37°C with shaking. Each was lawned, following the protocol listed previously, and 10 uL of phage lysate (at titer 10^9^ phage per mL) was spotted on each lawn. Each phage lysate was spotted onto each *E*. *coli* strain a minimum of 4 times (technical replicates). In addition to assessing the host range for the induced urinary phages, two other urinary coliphages previously isolated by our group [41] as well as non-urinary coliphages were spotted on urinary and laboratory *E*. *coli* strains. These coliphages include *Escherichia* phage Greed and *Escherichia* phage Lust, both isolated from urobiome samples, and non-urinary coliphages K30, P22, T2, T3, T6, T7 and N4 obtained from the Félix d’Hérelle Reference Center for Bacterial Viruses (Quebec City, Quebec Canada). Phages T2 and N4 were further tested on urinary *E*. *coli* strains collected from UTI-positive females (**S3 Table**). Plaques of T2 and N4 spots were confirmed via PCR of the plaque. T2 primers: 5’-aaacaggtgcctttggtgtc-3’ and 5’-ccacaatacccgcttcagtt-3’; N4 primers: 5’-tgctcttgataccagaggcaatg-3’ and 5’-tacgttggttcaacttcttggtt-3’. Primers were synthesized by Eurofins Genomics LLC. For all phages producing plaques for the host range spot assays, infection was again tested by harvesting the plaque and replating using the pour plates technique previously described.

## Results

### Prophages in urinary *E*. *coli* genomes

904 urinary *E*. *coli* draft and complete genome sequences were obtained from NCBI and screened for the presence of prophage via PHASTER [43]. Sixty-nine of these genome assemblies are of strains from our own collection. Of the 8,452 predicted prophage sequences found, 3,038 prophages were identified as intact, 1,508 as questionable, and 3,906 as incomplete. We focused our analysis on those identified as intact. 45 of the 904 genomes examined did not contain any intact prophages, while the majority (95%) of the urinary *E*. *coli* genomes harbored at least one intact prophage (**S1 Table**). 1,807 (59.48%) of the intact prophage sequences contained an identifiable integrase gene sequence (**S1 Table**). Each intact prophage sequence was compared to all annotated phage genome sequences to predict their taxonomy (**Table 1**).

**Table 1:** Taxonomic classification of predicted prophages in urinary *E*. *coli* based on blastn sequence similarity to sequenced phages.

Taxonomy	# Predicted Prophages
Myoviruses	788
Podoviruses	113
Siphoviruses	738
Unclassified Caudovirales	44
Unclassified bacterial viruses	20
Unknown	1,335

Virulence factor genes were identified in 25% (n = 765) of the intact coliphage sequences. These virulence-carrying intact prophage sequences were harbored by 710 of the 904 *E*. *coli* genomes examined. The most frequently identified virulence factor gene was *aaiQ* (n = 137), a pseudogene that has been linked to enteroaggregative *E*. *coli* pathogenesis [59]. The iron uptake *sit* operon was the next most prevalent virulence factor identified in 61 of the intact prophage sequences. When screened for antibiotic resistance associated genes, 299 genes were identified. These genes were found in 178 different intact prophage sequences (5.86%) from 165 different *E*. *coli* genomes. The most frequently identified antibiotic resistance gene was the ethidium multidrug resistance protein E (*emrE*), found in 47 of the prophage sequences. The transcription factor *marA* was the second most frequent antibiotic resistance associated gene found; 37 intact prophage sequences encoded for *marA*. While most strains had just one or two antibiotic resistance genes, *E*. *coli* Combat11I9 (GCF_002952095.1) includes one prophage sequence with 14 antibiotic resistance genes. **S1 Table** lists information about the virulence factors and antibiotic resistance associated genes found within the prophage sequences.

**Fig 1:**
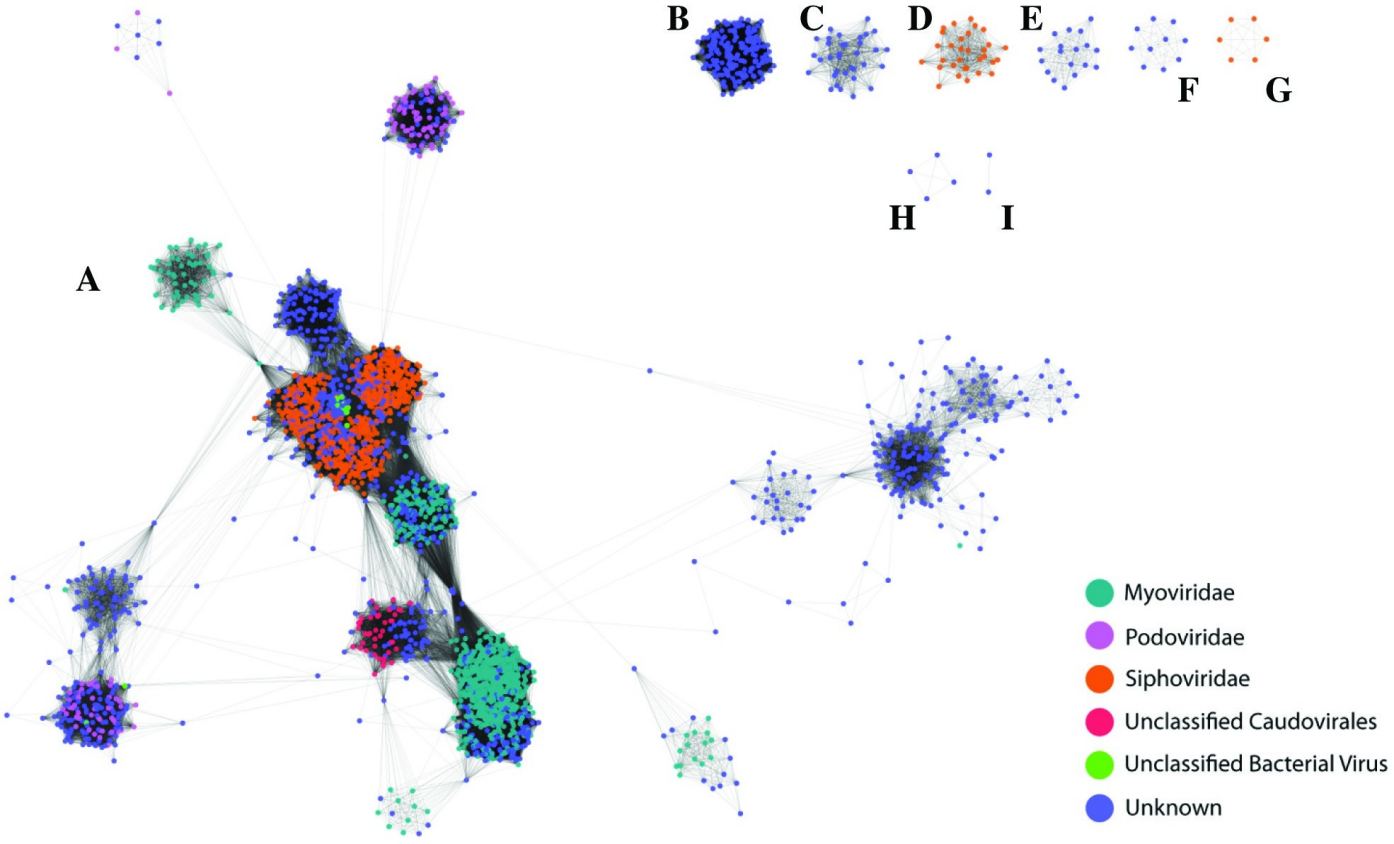
Diversity of urinary *E*. *coli* prophages. Each node corresponds with a predicted intact coliphage. Edges connecting nodes represent shared gene content. Individual connected components, clusters of prophages that share gene content, are indicated by letters A through I.

Intact prophages were annotated and the number of homologous genes between each prophage was calculated. Prophage similarity was assessed using a network approach in which nodes in the network represent a single prophage. Two nodes are connected by an edge in the network if they share a homologous gene sequence. We introduced a threshold for these edges such that only edges in which the two prophages (nodes) shared at least 30% of their genic content were visualized (see Methods). 3,025 of the 3,038 predicted prophages met this threshold, connected by 414,291 edges. **Fig 1** displays the network representation of the urinary prophages.

Nodes, representative of prophages, have been colored in this network according to their predicted taxonomic family. Over half of the identified prophages most closely resembled tailed phages (order Caudovirales), including the families Myoviridae, Podoviridae, and Siphoviridae. However, 43.9% of the predicted intact prophage sequences in the urinary *E*. *coli* genomes did not have significant similarity to previously isolated and sequenced phages. Our analysis of the shared genetic content of the predicted urinary prophages reveals nine connected components (**Fig 1**, labeled A-I). The individual connected components do not generally share homologous genes (see Methods). While the largest connected component, labeled A in **Fig 1**, includes the majority of Caudovirales, our network contains two distinct connected components of siphoviruses (**Fig 1D and 1G**, orange); these are separate from the siphoviruses within the primary (largest) connected component. There also are six distinct connected components of prophages for which we were unable to predict their taxonomic lineage (shown in **Fig 1B, 1C, 1E, 1F, 1H and 1I** in blue for “Unknown”).

Next, we investigated the network of prophages to identify clusters of similar prophage sequences, i.e., closely related prophages likely representative of the same species/strain. In total, 1,542 unique clusters of prophage sequences were identified (**S4 and S5 Tables**). The size of these clusters varied from singletons, i.e., single representative prophage sequences (n = 1,115, 72.31%) to a single cluster with 127 representatives (length ~43–50 Kbp; 87.73% nucleotide sequence identity between members). The consensus sequence of this large cluster was queried against the nr/nt Viruses database, identifying the top hit as a MAG sequence from the human metagenome (GenBank Accession No.: BK034715.1; 76% query coverage and 99.98% identity). Most of the clusters were small; 95% of the clusters had 5 or less prophage sequences. Four of the clusters had two prophage sequences identified from the same genome sequence. While two of these were the only instances of these prophage sequences in the data set, the other two were found in other genomes, including the cluster of 127 representatives. There is no association between cluster size and prophage sequence length (**S5 Table**). Sequences for the cluster representative are provided in **S1 File**.

### Comparison to gut *E*. *coli* prophages

Given prior evidence of UTIs by colonization of *E*. *coli* from the gut, we investigated whether the same or similar phage populations were harbored by urinary *E*. *coli* strains and phages from the gut. The 3,038 intact urinary prophages were screened against the phage database of the gut microbiome. These gut microbiome phage sequences include phages identified from metagenomic studies and, as such, include phages infectious of bacteria other than *E*. *coli*; furthermore, the host genus or species for most of these phage sequences is not known. As a result of this comparison, we found 2,006 urinary coliphages that exhibited ≥ 50% query coverage and ≥ 80% nucleotide sequence identity to phage sequences from the gut. This is representative of 863 of the 1,542 unique clusters of urinary prophages, and 560 of these clusters only had one representative sequence from the urinary coliphages. Furthermore, 117 intact urinary prophages, representative of 49 unique clusters, had a query coverage of 100% to a gut phage sequences (≥ 96% sequence identity) and 21 were identical (100% query coverage and sequence identity) to gut phage sequences (**S6 Table**). Thirteen of these 21 prophage sequences shared no significant sequence similarity to an annotated phage genome sequence. Thus, no taxonomic classification could be assigned. These 21 prophages sequences were also screened for antibiotic resistance genes and virulence factors, although none were found. The 21 identical prophages represent 12 unique clusters, including small clusters with just 2 members as well as the largest observed cluster with 127 representatives.

### Inducing urinary *E*. *coli* prophages

Nine urinary *E*. *coli* strains in our collection were selected for induction assays. These strains were selected as they were predicted to contain prophage sequences from singleton clusters, i.e., the urinary strain was the only genome predicted to contain this prophage, as well as prophage sequences from larger clusters, including clusters with prophages from *E*. *coli* strains from collections other than our own. For those prophages in singleton clusters, induction would lay the groundwork for future characterization of this phage strain. For those prophages in larger clusters, induction would provide insight into the putative temperance of the phage across many different urinary *E*. *coli* strains.

Using changes in pH, 10 induced prophages were identified as they were efficient in completely lysing one or more of the naïve laboratory strains tested–*E*. *coli* B, *E*. *coli* C, and *E*. *coli* K-12. We were able to identify the prophage that was induced via PCR (see Methods). Eight of the induced prophage sequences included recognizable integrase genes; i527 and i9930-2 did not. i6721 and i6653 were induced at all three pH conditions tested, pH = 4, 7 and 9; the phages isolated from pH = 7 were used for subsequent testing. The closest characterized and sequenced phage record in GenBank was identified for each of the induced prophage sequences (**Table 2**). Upon further inspection, it was found that the induced prophage from UMB9344 (i9344) is identical–query coverage and sequence identity = 100%–to one of the two induced prophages from UMB9930, i9930-1. The induced prophages from UMB5563 (i6653) and UMB6721 (i6721) also were similar, belonging to the same cluster (**S7 Table**). A visualization of the induced prophage genomes is provided in **S1 Fig**.

**Table 2:** pH-induced prophages from urinary *E*. *coli* strains and their closest characterized and sequenced phage. Phages are named as i plus the *E*. *coli* strain number from which they were induced. As two phages were isolated from *E*. *coli* UMB9930, they are signified as “-1” and “-2”.

Phage ID	pH Condition (*E*. *coli* host)	Predicted Product Amplified by PCR	Closest Blast Hit			
			Description	Accession No.	Query Coverage	Sequence Identity
i527	4 (*E*. *coli* C)	DUF2560 family protein	Enterobacteria phage CUS-3	CP000711.1	60%	96.04%
i6653	4, 7 and 9 (*E*. *coli* C)	Phage terminase, endonuclease subunit GpM	Bacteriophage L-413C	AY251033.1	77%	97.80%
i6721	4, 7 and 9 (*E*. *coli* C)	Phage terminase, endonuclease subunit GpM	Enterobacteria phage fiAA91-ss	NC_022750.1	75%	93.48%
i9006	4 (*E*. *coli* C)	Phage replication protein GpA, endonuclease	*Escherichia* virus P2_4E6b	NC_049389.1	79%	96.52%
i9105	7 (*E*. *coli* C)	Phage tail fiber protein GpH	Bacteriophage L-413C	AY251033.1	88%	99.97%
i9208	4 (*E*. *coli* C)	Phage tail fiber protein GpH	*Escherichia* phage pro147	KR073660.1	77%	96.87%
i9344	4 (*E*. *coli* C)	Phage major tail tube protein GpFII	*Escherichia* virus P2_4C9	NC_049388.1	73%	96.24
i9346	7 (*E*. *coli* C)	Phage lysis regulatory protein, LysA	Bacteriophage R18C	NC_049461.1	68%	97.31%
i9930-1	7 (*E*. *coli* C)	Phage baseplate assembly protein GpJ	*Escherichia* virus P2_4C9	NC_049388.1	73%	96.24
i9930-2	7 (*E*. *coli* K-12)	Phage head, portal protein B	Stx-1a-converting phage Stx1_499	LC567825.1	96%	94.88%

### Phage host range

Each induced urinary coliphage was tested for its ability to completely lyse laboratory strains of *E*. *coli*, as well as urinary *E*. *coli* strains representative of the diversity of *E*. *coli* phylotypes found within the female bladder [7]. In parallel, we tested two lytic urinary siphoviruses previously isolated by our group [41], *Escherichia* phage Greed and *Escherichia* phage Lust, alongside several well-studied lytic coliphages routinely used in the laboratory—*Escherichia* phage K30, Enterobacteria T3 and Enterobacteria T7 (family Autographiviridae), *Salmonella* virus P22 (family Podoviridae), Enterobacteria phage T2 and T6 (family Myoviridae), and *Escherichia* virus N4 (family Schitoviridae). The results of these host range assays are shown in **Table 3**.

**Table 3:** Phage efficacy of lysing laboratory and urinary bacteria. If a phage completely lysed the *E*. *coli* strain, the table lists “+”. The phylotype for each urinary *E*. *coli* strain is listed [7]. *E*. *coli* UMB0103 was isolated from a female with OAB symptoms; the other urinary *E*. *coli* strains were isolated from females with UTI symptoms.

	LABORATORY *E*. *COLI* STRAINS			URINARY *E*. *COLI* STRAINS									
				A	B1	B2				D			F
	B	C	K-12	1358	1180	1195	5924	1220	1162	1225	1337	7431	103
**Induced Phages**													
**i527**		**+**					**+**	**+**		**+**			
**i6653**	**+**	**+**	**+**				**+**			**+**			
**i6721**		**+**		**+**				**+**	**+**	**+**			**+**
**i9006**		**+**											
**i9105**		**+**								**+**			
**i9208**		**+**	**+**				**+**			**+**			
**i9344**		**+**	**+**				**+**			**+**			
**i9346**		**+**								**+**			
**i9930-1**		**+**	**+**				**+**			**+**			
**i9930-2**		**+**	**+**				**+**						
**Urinary Phages**													
**Lust**	**+**	**+**	**+**				**+**	**+**	**+**				
**Greed**	**+**	**+**	**+**				**+**	**+**	**+**			**+**	
**Laboratory Phages**													
**K30**	**+**	**+**						**+**	**+**	**+**			
**P22**	**+**					**+**		**+**				**+**	
**T2**	**+**	**+**	**+**	**+**	**+**		**+**	**+**	**+**	**+**		**+**	**+**
**T3**	**+**	**+**	**+**			**+**	**+**	**+**	**+**			**+**	**+**
**T6**	**+**	**+**	**+**		**+**	**+**	**+**	**+**	**+**			**+**	**+**
**T7**	**+**	**+**	**+**			**+**	**+**	**+**				**+**	
**N4**		**+**	**+**	**+**	**+**	**+**		**+**	**+**	**+**	**+**		**+**

The well-studied phages T2 and N4 were most efficient in completely lysing *E*. *coli* strains isolated from UTI patients. Each was capable of completely lysing eight of the ten urinary strains tested. Given this success, we further tested T2 and N4 against an additional 103 *E*. *coli* strains isolated from urine samples from females with UTI symptoms. A list of the strains tested, including serotype and phylotype, are listed in **S3 Table**. Sixty-three strains were completely lysed by T2, 71 strains were completely lysed by N4, 48 strains were completely lysed by both T2 and N4, and 17 strains were not completely lysed by either phage (**Table 4**). Thus, 83.5% of the *E*. *coli* strains tested from females with UTI symptoms would be susceptible to a phage cocktail containing T2 and N4.

**Table 4:** Susceptibility of *E*. *coli* strains from females with UTI symptoms to bacteriophages T2 and N4.

	# of *E*. *coli* lysed	% of *E*. *coli* lysed
Both T2 & N4	48	46.6%
N4 only	23	22.3%
T2 only	15	14.6%
Neither	17	16.5%

## Discussion

While our prior catalog of prophages in urinary bacteria considered just four *E*. *coli* strains [28], here we have evaluated the incidence of lysogeny among a significantly larger (n = 906) sampling of urinary *E*. *coli* strains. While all four of the genomes examined in our prior study were found to harbor prophage sequences, only one was predicted to include an intact prophage [28]. Here, the majority (95%) of the urinary *E*. *coli* genomes harbored at least one intact prophage sequence indicating that lysogeny is prevalent among *E*. *coli* strains of the urobiome. While some (~25%) of these prophage sequences carried virulence factor genes, very few encoded antibiotic resistance genes (**S1 Table**). One of the lysogen-associated proteins, the integrase, was identified in many (59.48%) of these intact prophage sequences. Those lacking the integrase necessitate further investigation to ascertain if they are temperate phages or prophage relics.

We found that only 56.1% of the predicted intact prophage sequences resembled characterized phage sequences. The remaining prophage sequences either represent novel prophages or highly mosaic prophages infectious of *E*. *coli*. This concurs with our prior examination of genomes of other taxa from the urobiome, which found a high percentage of unknown, novel prophages [28]. It is important to note that the intact prophages examined here do not include any representatives of inoviruses. Current prophage prediction tools–including PHASTER–frequently fail to identify inovirus sequences [60–62]. Previously, we identified an inovirus harbored by Enterobacteriaceae including urinary *E*. *coli* strains [63]. Inovirus sequences could be included in the questionable and/or incomplete prophage sequence predictions excluded from our analysis. We thus hypothesize that the intact prophage sequences examined here underestimates the genetic diversity of prophages within urinary *E*. *coli* strains. It does, however, provides a good approximation of the diversity of tailed phages infectious of urinary *E*. *coli*.

Our network-based analysis of the shared genetic content between the predicted urinary prophages identified nine connected components (**Fig 1**). The largest connected component contains the majority of the tailed phages identified. From the network, we can posit taxonomic classification of “Unknown” (**Fig 1**, blue) prophages within this connected component as members of Caudovirales. It is important to note that our network analysis includes a threshold for the minimum percentage of gene content shared for an edge to be drawn. Thus, edges represent “modules” shared between prophages. When this threshold is removed, such that every homologous gene shared between two prophages (nodes) is represented by an edge, all prophages are connected producing a single connected component. Previous analyses of phage genomes have taken a similar modular approach [64, 65]. In this prior work, modules in temperate phages were found to correspond to functional modules [64]. These modules often serve as markers corresponding to the evolutionary history of related phages [66].

Of the 1,542 unique clusters of urinary prophage sequences identified, many (55.97%, n = 863) shared sequence similarity to phages identified in the gut microbiota suggesting that similar coliphages infect *E*. *coli* strains found in both niches. The interconnectedness of the urobiome and gut microbiota is an open question. Prior studies have shown that *E*. *coli* causing UTIs can come from the gastrointestinal tract [2–5]. However, a previous examination of the bacterial constituents of the gut and urinary tract did not find the two niches to be connected [67]. To date, the interconnectedness of the phage populations between these two microbiota have not been examined. The similarities observed here serve as the impetus for future studies.

Our ability to induce prophages from the urinary *E*. *coli* strains supports the working hypothesis that the PHASTER predictions of intact prophages are temperate phages, rather than prophage relics. Changes in pH were able to stress the bacterial cell and trigger the induction process. Changes in pH are particularly relevant to the urinary tract and its microbiota. *Lactobacillus* species are dominant members of the healthy female bladder [8], and lactobacilli reduce the pH of the urogenital environment (see review [68]). Thus, fluctuations in lactobacilli abundances within the urinary tract could lead to changes in pH, which in turn could trigger induction. While nine out of the ten induced urinary prophages completely lyse the naïve laboratory strain *E*. *coli* C, they have varied abilities to completely lyse urinary *E*. *coli* strains. Two main observations can be made from our host range assays (**Table 3**). First, no single induced urinary phage was capable of completely lysing all of the urinary *E*. *coli* strains tested. Second, induced urinary phages capable of completely lysing one representative of a phylotype were not necessarily able to completely lyse all of the strains tested for that same phylotype. While only a single strain from phylotype A, B1, and F were tested, multiple representatives of phylotypes B2 and D, the most common phylotypes in the bladder [7], were tested. It is well documented that phages often are infectious of some but not all strains of a given species (see review [69]). The observed lack of plaquing by these induced urinary phages could be the result of (1) the phage’s inability to infect the *E*. *coli* strain, (2) the phage’s inability to evade the host’s defenses (e.g., CRISPR/Cas system), (3) the phage’s inability to completely lyse the bacterial cell, (4) entrance of the temperate phage back into the lysogenic life cycle, and/or (5) bacterial resistance due to superinfection.

In contrast, the lytic laboratory coliphages were far more successful in completely lysing the urinary *E*. *coli* strains. They also were far more successful than the lytic urinary phages Lust and Greed. T2 and N4 were able to complete lyse at least one strain from all five phylotypes tested. The susceptibility of the urinary *E*. *coli* strains to these lytic laboratory coliphages may be due to the very fact that they are less likely to encounter these phages in the urinary tract than they are to encounter the induced urinary prophages. This suggests that phages that are not native to the urinary tract would be better candidates for use as phage therapies. Phage therapy is increasingly being explored for treatment of bacterial infections including *E*. *coli* and UTIs (see reviews [70–72]). Our further tests of T2 and N4 phages found that they were able to completely lyse most UTI-associated *E*. *coli* strains tested (**Table 4**), and thus should be explored for UTI treatments.

## Supporting information

S1 Table Urinary *E*. *coli* genomes retrieved from NCBI and screened for prophage sequences. (XLSX)

S2 Table PCR primers used to identify induced prophages. (XLSX)

S3 Table Urinary *E*. *coli* strains tested for susceptibility to T2 and N4. S3 Strains were either isolated from an individual with an acute urinary tract infection (UTI) or recurrent UTI (rUTI).
(XLSX)

S4 Table Prophage clusters identified in each urinary *E*. *coli* genomes. (XLSX)

S5 Table Cluster sizes and length of the representative sequence for the cluster. (XLSX)

S6 Table Urinary prophages identical to gut phage sequences. (XLSX)

S7 Table Cluster information for induced prophages. (XLSX)

S1 Fig Annotations of induced prophages. (PDF)

S1 File Nucleotide sequences of the cluster representatives. (FASTA)
