# Supplementary figures and images for: Coliphages of the human urinary microbiota
Source: PLoS One. 2023 Apr 13;18(4):e0283930. doi: 10.1371/journal.pone.0283930 (PMC10101464; doi:10.1371/journal.pone.0283930)

### S1 Fig. Annotations of induced prophages.

i527

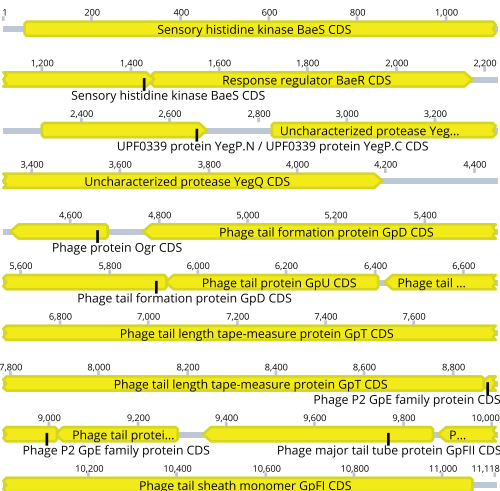

## i6653 & i6721

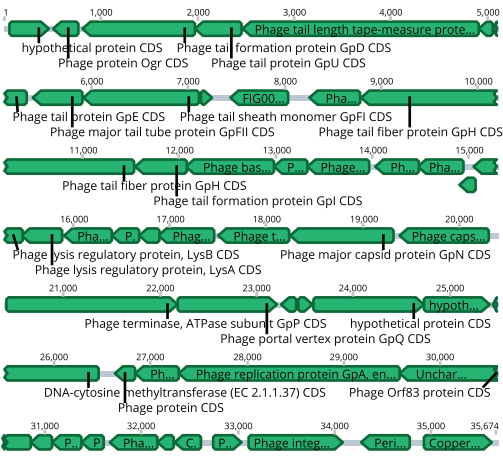

i9006

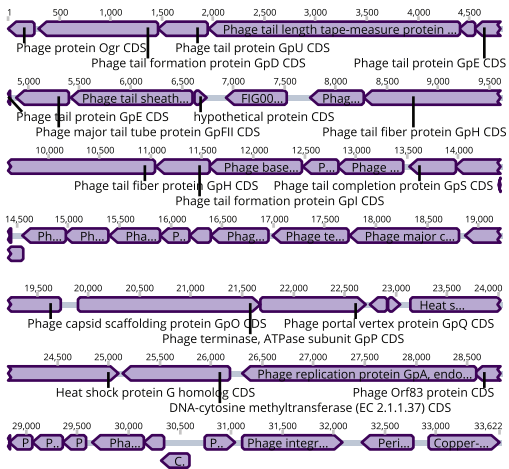

i9105

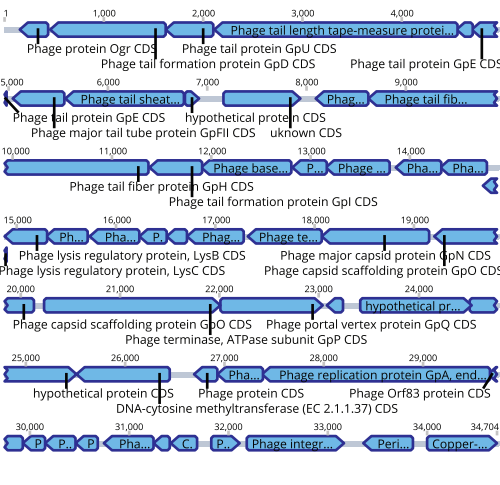

i9208

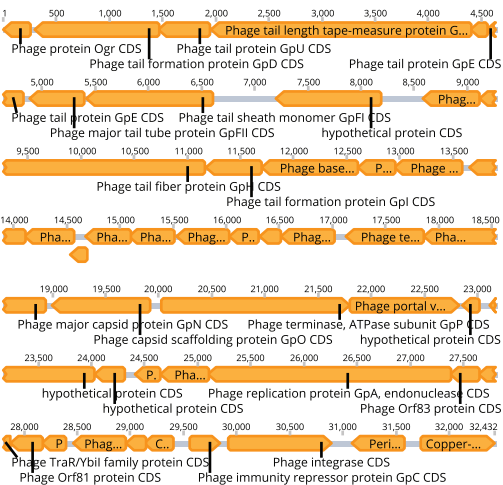

i9344 &amp; i9930-1

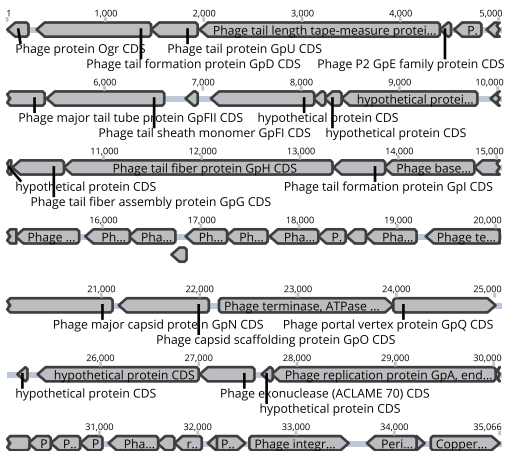

i9346

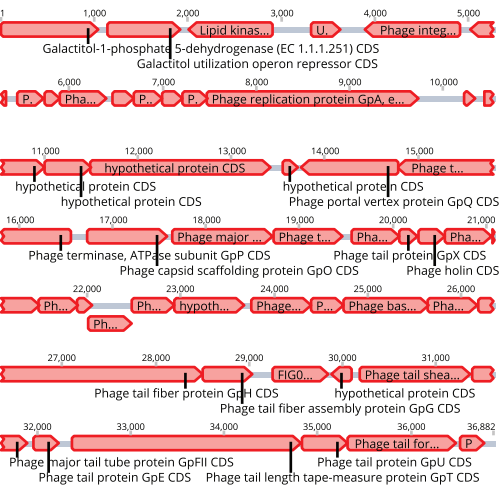

i9330-2

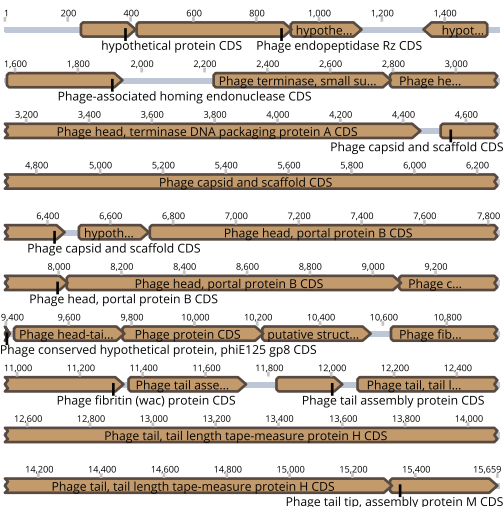

Supplement: S1 Fig — (PDF) [file pone.0283930.s008.pdf]
